# *Klebsiella pneumoniae* liver abscess with purulent meningitis and endogenous endophthalmitis: A case report

**DOI:** 10.3389/fsurg.2022.894929

**Published:** 2022-08-24

**Authors:** Fang Li, Wenfei Zheng, Jian Yu, Linyan Zhao

**Affiliations:** ^1^ Department of Stroke Center, Dalian Municipal Central Hospital, Dalian, China; ^2^ Department of Critical Care Medicine, The Second Affiliated Hospital of Dalian Medical University, Dalian, China

**Keywords:** *Klebsiella pneumoniae* liver abscess, meningitis, hypervirulent *Klebsiella pneumoniae*, endophthalmitis, diabetes mellitus

## Abstract

This is a rare but typical case of a *Klebsiella pneumoniae* liver abscess with migratory infections including purulent meningitis and endogenous endophthalmitis. The patient had a chief complaint of 7 days of fever, 4 days of blurry vision, and 4 h of glossolalia. Ultrasound scan and computed tomography (CT) suggested a liver abscess. Both blood and drainage fluid cultures grew *K. pneumoniae* with a high mucosal phenotype. The patient was finally diagnosed with a *K. pneumoniae* liver abscess, purulent meningitis, and endogenous *K. pneumoniae* endophthalmitis in the right eye. Ultrasound-guided percutaneous catheter drainage (PCD) of the liver abscess was performed, and meropenem was used to control infection. The patient was given 0.1 ml of vancomycin (10 g/L) and 0.1 ml of ceftazidime (20 g/L) were by intravitreal injection for the treatment of endophthalmitis. The infection was gradually controlled after such treatments. The patient was discharged from our hospital with an improved condition. However, during the time of follow-up, she developed complications due to severe pneumonia and eventually died in a local hospital. This case revealed that a rapid diagnosis followed by appropriate treatment would improve prognosis and prevent severe metastatic complications.

## Introduction

*Klebsiella pneumoniae* is a common bacterium in nosocomial infections, which is usually seen in pneumonia, urinary tract infections, abdominal infections, surgical wound infections, and concurrent bacteremia (1). In 1986, Taiwan reported a *K. pneumoniae* that can cause abscesses at multiple sites, which was first defined as hypervirulent *K. pneumoniae* (hvKP) (2). Subsequently, cases were reported in other regions such as Korea, the United States, Canada, France, Europe, South Africa, and Australia, but infections occurred mainly in Asia (3). In recent years, the increasing number of *K. pneumoniae* liver abscess (KLA) cases worldwide has become an important clinical topic. This report presents a case of KLA in an intensive care unit in mainland China.

## Case description

A 69-year-old woman consulted a hospital with a chief complaint of 7 days of fever, 4 days of blurry vision, and 4 h of glossolalia. She had a history of untreated diabetes mellitus and tuberculosis, which was cured 50 years ago. She had no other medical history and was not an alcohol drinker or a smoker. On admission, her initial vital signs included a body temperature of 38.2 °C, a heart rate of 118 beats/min, a blood pressure of 128/60 mmHg, a respiratory rate of 20 breaths/min, and oxygen saturation of 98%. She was experiencing clouding of consciousness and was not able to cooperate with physical examination. After opening her eyelid, a mild conjunctival and corneal edema was found. Massive fibrinous exudation showed at the margin of the pupil with no pupillary light reflex in the right eye. There was percussion pain in the hepatic region and tenderness in the right upper quadrant of the abdomen. There was no hepatosplenomegaly. Meningeal irritation signs were suspected to be positive.

Laboratory test results revealed a white blood cell count of 17.5 × 10^9^/L, with a neutrophil predominance of 89.8%, and a platelet count of 128 × 10^9^/L. The concentration of C-reactive protein (CRP) was 204.87 mg/L, and the procalcitonin (PCT) level was 14.82 ng/L. Arterial blood gas results were as follows (on 6 L/min oxygen *via* nasal cannula): a pH of 7.40, PaCO_2_ of 15 mmHg, PaO_2_ of 96 mmHg, HCO_3_ of 9.4 mmol/L, and lactic acid of 1.9 mmol/L. The coagulation profile showed an international normalized ratio of 1.47, an activated partial thromboplastin time of 38.9 s, and a prothrombin time of 14.9 s. Liver function test results were as follows: aspartate aminotransferase of 33.8 U/L; alanine aminotransferase of 33 U/L; lactate dehydrogenase of 303.86 IU/L; total bilirubin of 10.55 µmol/L; and direct bilirubin of 6.04 µmol/L. The fasting blood sugar level was 14.5 mmol/L, and HbA1C was 13.3%. A urine full report showed proteinuria (++) with 1.3 pus cells per high power field. Creatinine was 87 µmol/L, and urea was 8 mmol/L. Peripheral blood cultures were collected twice.

**Figure 1:**
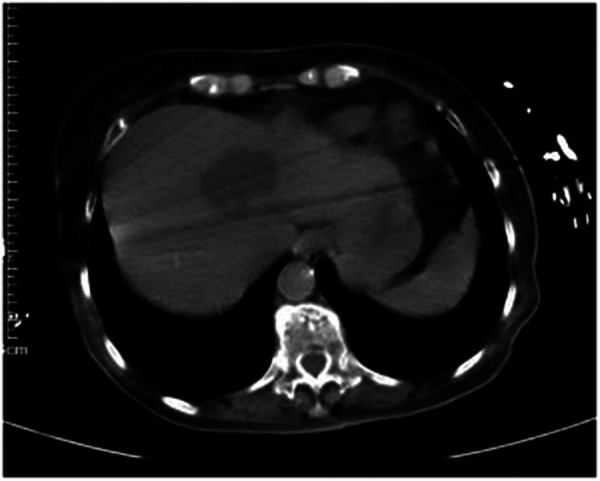
CT on admission (45 mm × 38 mm).

**Figure 2:**
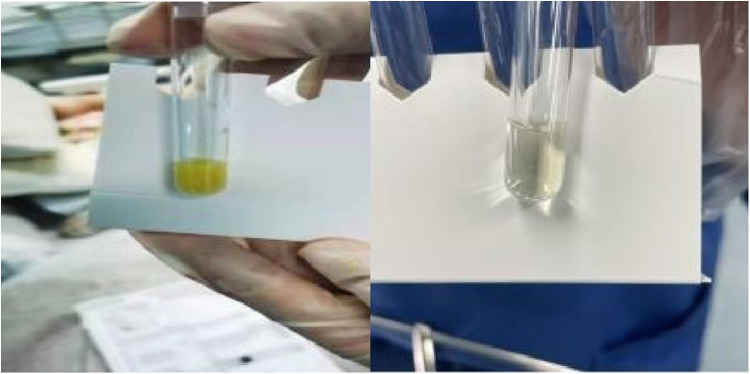
Cerebrospinal fluid (CSF) before treatment and after treatment.

Further imaging examinations were performed. An ultrasound scan (US) revealed a mixed echogenic mass containing solid and cystic components in the right lobe of her liver, measuring 5.0 × 3.9 cm in size. An abdominal CT scan showed a single low-density focus in the right lobe of the liver (45 mm × 38 mm), which demonstrated a possible liver abscess (Figure 1). No other intra-abdominal pathologies such as gallstones were observed on these imaging examinations. In order to detect intraocular diseases, an ocular ultrasound was performed. It revealed lens clouding in both eyes and vitreous clouding in both eyes (excluding blood or pus accumulation in the vitreous of the right eye), excluding partial retinal detachment in the right eye. Because of her 4-h delirium before admission, meningitis was considered. A head CT scan and a lumbar puncture were necessary. However, there were no remarkable findings according to the head CT result. Puncture examination showed that the intracranial pressure was 150 mm H_2_O. The cerebrospinal fluid (CSF) appeared yellow and purulent (Figure 2), revealing a 580,000 × 10^6^/µL white blood cell count with a multinucleated cells percentage of 98%, protein above 15 g/L, and glucose less than 1.1 mmol/L. The CSF was also submitted for Gram staining and bacterial culture.

The patient was preliminarily diagnosed with a primary liver abscess, endogenous endophthalmitis, and meningitis. Ultrasound-guided percutaneous catheter drainage (PCD) of the liver abscess was immediately performed, with a continuous drainage of 110-ml yellow pus. Specimens were retained for culture. Meropenem was given intravenously as an empirical treatment. The patient was given 0.1 ml of vancomycin (10 g/L) and 0.1 ml of ceftazidime (20 g/L) were by intravitreal injection. On the third day after admission, the oxygenation index reduced to 180, and the self-cleaning ability of the airway was poor. A tracheotomy was performed, and mechanical ventilation was started at once. Both blood and drainage fluid cultures grew *K. pneumoniae* with a high mucosal phenotype, which was sensitive to all the tested antibiotics. A string test showed a positive result (string ≥5 mm). The cerebrospinal fluid culture showed gram-negative bacteria. With the completion of the assays, the ocular disease was diagnosed as endophthalmitis complicated by a liver abscess caused by hypervirulent *K. pneumoniae*. Ceftazidime was used alone for intravitreal injection.

In conclusion, the patient had a liver abscess as the primary focus of infection with migratory infection of the eye and meninges. The pathogen was considered to be hypervirulent *K. pneumoniae* (hvKP) according to the results of the drug sensitivity test, clinical manifestations, and the string test (+). The patient was finally diagnosed with a *K. pneumoniae* liver abscess, purulent meningitis, and endogenous *K. pneumoniae* endophthalmitis in the right eye. Meropenem was continued to control the bloodstream infection. After 15 days of treatment, her body temperature became normal, and the rechecked blood routine, CRP, and PCT had normalized. A lumbar puncture and an abdominal CT were performed as part of reexamination. The CSF appeared transparent (Figure 2), revealing a 34 × 10^6^/µl white blood cell count with a multinucleated cells percentage of 5%, protein of 3 g/L, and glucose of 6.1 mmol/L. CT showed a low-density shadow (15 mm × 21 mm) that was much smaller than the earlier one in the right lobe of her liver. She was successfully weaned from the ventilator with an oxygenation index of beyond 300 and was catheterized with oxygen at the tracheotomy site. She opened her eyes spontaneously but was not able to cooperate with the physical examination. Therefore, the eyesight of her right eye could not be evaluated. She had stable vital signs with a heart rate of 94 beats/min, blood pressure of 132/75 mmHg, and oxygen saturation of 100%. The anti-infective therapy was changed from meropenem to ceftazidime and lasted for 2 weeks. The patient was discharged with an improved condition and was followed up for long-term outcomes. However, during follow-up, she developed complications due to severe pneumonia and eventually died in a local hospital.

## Discussion

Unlike classical *K. pneumoniae* (cKP), hvKP is highly viscous and virulent and can cause community-acquired infections, including a septic liver abscess, pneumonia, meningitis, and endophthalmitis. Most of the hypervirulent strains express a hypermucous phenotype, which is defined by a string test. When a standard bacteriological loop is passed through a colony, mucous strings formed by strains are greater than 5 mm after cultivation for 18–24 h on an agar plate (4). However, it has been shown that not all strains are highly viscous. So far, there has been no specific examination to distinguish between hvKP and cKP, so it is more reliable to combine a string test with clinical manifestations to make a confirmed diagnosis.

A *K. pneumoniae* liver abscess (KLA) is a liver abscess without predisposing factors and hepatobiliary system diseases, which is almost always caused by *K. pneumoniae*. Most cases of community-acquired KLA were reported from Asia, with Taiwan having the highest number of reported cases (5). KLA can be accompanied by extrahepatic invasive manifestations, with the lung, brain, and eye being the most common sites of invasion. Therefore, liver abscesses with extrahepatic complications such as endophthalmitis and meningitis are called invasive *K. pneumoniae* liver abscess syndrome.

The pathogenesis of invasive liver abscess syndrome includes bacterial virulence factors and host factors. Bacterial virulence factors consist of a capsule, including a high mucosal phenotype of hypervirulent strains; a lipopolysaccharide (LPS); siderophores; and fimbriae. The hypermucous phenotype and a greater expression of siderophores may be closely related to the high virulence of hvKP (6). Based on the diversity of the polysaccharide components of the capsule and different structures and antigens, *K. pneumoniae* can be divided into at least 79 capsule serotypes (7). hvKP contains eight serotypes, namely, K1, K2, K5, K16, K20, K54, K57, and KN1, of which K1 and K2 are the most prevalent serotypes identified in monomicrobial *K. pneumoniae* liver abscesses (8, 9). Prevalent serotypes vary by ethnic groups: K1 is the predominant serotype in Asia, while K2 is more common in Europe and North America (10). Unfortunately, we could not determine the serotype of this patient because our institution was unable to measure the serotype due to technical limitations. Hypervirulent strains make a hypercapsule *via* greater production of capsular material, which shows enhanced resistance to the host immune response, including inhibiting the proximity of antibodies and antimicrobial peptides and reducing complement killing and phagocytosis by human neutrophils and macrophages. RmpA/RmpA2, the major activating transcription factor of *K. pneumoniae* capsule, promotes the expression of *K. pneumoniae* capsule genes and is closely associated with the high virulence and high mucosal phenotype of hvKP (11). Also, the ability to obtain iron is critical to the growth and reproduction of bacteria. According to a recent study, hvKp strains are capable of producing larger and more activated iron-absorbing molecules as compared to cKP. The increased capacity of uptaking iron makes hvKP more resistant to complement killing, which may lead to their virulence and pathogenicity (12).

In addition to impaired host immunity, KLA is significantly associated with diabetes mellitus (DM) or impaired glucose tolerance, especially in patients with hematogenic metastatic infections. Endogenous *K. pneumoniae* endophthalmitis (EKE) is a common complication in KLA accompanied by DM (13). Having diabetes and being >65 years were independent predictors of ocular pyogenic infection or central nervous system (CNS) complications in patients with liver abscesses (14). The patient of this case had a host factor of diabetes mellitus. The bacteria culture and string test indicated that the pathogen was hypervirulent *K. pneumoniae* with a high mucosal phenotype. Both are consistent with the pathogenesis of invasive *K. pneumoniae* liver abscess syndrome.

Typical clinical manifestations of a liver abscess are fever and/or chills, followed by gastrointestinal symptoms (e.g., gastrointestinal upset, diarrhea, vomiting, and nausea), and jaundice. The laboratory tests show leukocytosis, increased concentrations of CRP, and abnormal results of liver function tests. US generally presents a solid echogenic mass with irregular or indistinct margins (15). CT plays a key role in the diagnosis of a liver abscess. Abdominal CT manifests a solid, thin-walled, low-density focus without edge enhancement, which is usually located in the right lobe of the liver as compared to liver abscesses caused by other bacteria (16). KLA may be complicated by thrombophlebitis; thus, the presentation of CT needs to be distinguished from hepatocellular carcinoma with vascular thrombosis (17). The solid appearance may be related to the capsule serotype of the strain. There is not enough time for the parenchyma to break down completely into homogeneous pus owing to the rapid invasion of hypervirulent strains. A mixture of immature pus and debris may be produced, resulting in a lower amount of pus aspirated at the initial drainage than in other pyogenic abscesses (15). Studies have also reported that the rate of metastatic infection associated with KLA was higher than that associated with non-KLA (18). Metastatic infection generally occurs beyond the abdominal region at more distant locations, with a predominance of the eyes (EKE) and CNS (e.g., meningitis and brain abscess). Other manifestations include septic pulmonary embolism, lung abscess, splenic abscess, cervical abscess, psoas abscess, necrotizing fasciitis, spondylitis, and osteomyelitis (19).

Recently, the therapy for a liver abscess is drainage combined with appropriate antibiotics. Methods of drainage include percutaneous needle aspiration (PNA), US- or CT-guided PCD, laparoscopic drainage, and surgical drainage. The choice depends on the size and number of abscesses. PNA or PCD can be used for the treatment of small single abscesses (*d* ≤ 5 cm). Both procedures are probably equally effective and safe. PNA has the potential for repeated aspirations (20, 21). PCD fits large abscesses (*d* > 5 cm) better (22). Giant (*d* > 10 cm) or multiloculated abscesses are often treated inadequately and are more likely to lead to a recurrence (23). Indications to perform surgery include very large (>5 cm) or multilocular abscesses, unsuccessful percutaneous drainage, presence of intra-abdominal infection (peritonitis), and ruptured abscesses (24). In recent years, laparoscopic drainage has gradually become a better alternative than open surgery (25). In this case, the patient had a monolocular liver abscess with thick pus; thus, PCD was considered more appropriate.

The selection of antimicrobial therapy should be based on *in vitro* susceptibilities and clinical effects. The specific therapy depends on the patient's condition (e.g., history of drug allergy, history of antibiotic usage, drug toxicity, interactions, and opportunity for use). Clinical isolates are usually of high sensitivity to β-lactam antibiotics. Cephalosporin is the main choice for the treatment of KLA in Asia (26). Compared with a first-generation cephalosporin, a third-generation cephalosporin manifests a better clinical efficacy with a medication time of 2–4 weeks for a solitary abscess and 6 weeks for multiple abscesses (27). The specific course of treatment is determined from the patient's imaging examinations, inflammatory indicators, and clinical presentation. Without the production of extended-spectrum β-lactamases (ESBL), third-generation cephalosporins seem to be a proper choice for *K. pneumoniae* meningitis due to their better penetration into the cerebrospinal fluid (28). When ESBL strains are suspected, imipenem and meropenem can be used as an anti-infective therapy instead of the third-generation cephalosporins (29). Both intravitreal and intravenous injections are necessary for endophthalmitis, and sometimes, a vitrectomy is required for local treatment. The mainstay of intravitreal antibiotics is third-generation cephalosporins, vancomycin, and aminoglycoside (30). In this case study, we determined that the therapy depends on the final diagnosis and medication history: using meropenem for 2 weeks, followed by triple cephalosporin for 2 weeks. In this patient, vitreous fluid was restrained for culture, but it grew no bacterium. The reason for this was considered to be either the use of antibiotics or the low culture-positive rate of *Klebsiella pneumoniae* itself. Both blood and drainage fluid cultures grew *K. pneumoniae*. Also, a liver abscess caused by hypervirulent *K. pneumoniae* can be accompanied by extrahepatic invasive manifestations, with the lung, brain, and eye being the most common sites of invasion. All of the above revealed that endophthalmitis was caused by *Klebsiella pneumoniae*. Therefore, ceftazidime was used for intravitreal injection.

## Conclusion

In this report, we describe a case of a *K. pneumoniae* liver abscess with endogenous *K. pneumoniae* endophthalmitis and purulent meningitis. Manifestations of the invasive syndrome and a positive result of the string test can be the first clinical clues. A rapid diagnosis followed by appropriate treatment would improve a patient's outcome and prevent severe metastatic complications. Clinicians should increase their awareness of hvKP and provide appropriate treatments with an early diagnosis. Further investigation is needed to identify the source and confirm the pathogenic mechanism of hypervirulent *K. pneumoniae*.

## Data Availability

The original contributions presented in the study are included in the article/Supplementary Material, further inquiries can be directed to the corresponding author.
